# Personalized Kampo Medicine Facilitated Both Cytotoxic T Lymphocyte Response and Clinical Benefits Induced by Personalized Peptide Vaccination for Advanced Esophageal Cancer

**DOI:** 10.1155/2016/5929525

**Published:** 2016-09-15

**Authors:** Daisuke Muroya, Shigeru Yutani, Shigeki Shichijo, Akira Yamada, Shinjiro Sakamoto, Masayasu Naito, Koji Okuda, Michi Morita, Rin Yamaguchi, Kyogo Itoh

**Affiliations:** ^1^Department of Surgery, Kurume University School of Medicine, Kurume, Japan; ^2^Cancer Vaccine Center, Kurume University, Kurume, Japan; ^3^Research Center for Innovative Cancer Therapy, Kurume University School of Medicine, Kurume, Japan; ^4^Department of Molecular and Internal Medicine, Hiroshima University School of Medicine, Hiroshima, Japan; ^5^Department of Surgery, Nagasaki University Graduate School of Biomedical Sciences, Nagasaki, Japan; ^6^Division of Pathology, Medical Center of Kurume University, Kurume, Japan

## Abstract

We retrospectively evaluated if personalized Kampo medicine (PKM) could facilitate CTL responses and clinical benefits induced by personalized peptide vaccination (PPV), in which HLA-matched vaccines were selected and administered based on the preexisting host immunity, for advanced esophageal cancer (aEC) patients. Among 34 aEC patients entered in the clinical study, 23 patients received PKM and PPV without (*n* = 12) or with chemotherapy (*n* = 11), while the remaining 11 patients did not receive PKM but received PPV without (*n* = 6) or with chemotherapy (*n* = 5), respectively. Incidence of adverse events was significantly lower or higher in PKM and PPV arm (*n* = 23) or PPV and chemotherapy arm (*n* = 16) as compared to that of the counter arm (*n* = 11 or 18), respectively. Postvaccination PBMCs from the patients undergoing PKM and PPV showed significantly higher CTL responses as compared to the counter arm. The median progression-free survival (PFS) or median survival time (MST) of 34 patients was 2.9 or 7.6 months, respectively. The combination therapy in PPV and PKM arm, but not that in PPV and chemotherapy arm, significantly (*P* = 0.02) prolonged MST. These results could warrant a next step of prospective clinical study of PKM and PPV for aEC patients.

## 1. Introduction

The majority of esophageal cancer patients present with unresectable or metastatic disease at the time of diagnosis, and recurrences are common in these advanced esophageal cancer (aEC) patients [1–4]. Palliative treatment is the only option for controlling cancer-related symptoms in these patients. Furthermore, data are scarce on the use of second-line therapies for cases of relapse or refractoriness, and there remains no consensus on the optimal second-line treatment [1–4]. Therefore, newer therapeutic approaches for aEC should be developed, and immunotherapy would be a promising candidate. We have developed a novel regime of PPV, in which peptides are selected and administered based on the preexisting host immunity before vaccination [5–7]. PPV could provide both CTL responses and clinical benefits for advanced bladder cancers and colorectal cancers as reported [5–7]. However, PPV by itself could not generally provide either CTL boosting or clinical benefits for the majority of advancer cancer patients except for those two types of cancers, and thus the combination therapies, including salvage chemotherapy or targeted therapy, were required [5–7]. In addition to these combination therapies, the traditional Japanese Kampo medicine (KM) could be an attractive candidate since it could control cancer-related symptoms and maintain a good quality of life. Subsequently, we previously conducted a randomized clinical study to investigate whether the combined usage of juzentaihoto (JTT), one of Kampo medicines frequently used for cancer patients, could affect antigen-specific immunity in advanced pancreatic cancer patients undergoing PPV [8]. However, JTT neither affected CTL responses specific to the vaccine antigens nor prolonged overall survival, although it prevented the deterioration of patients' conditions. Then, to explore a new treatment modality for aEC patients, we retrospectively evaluated in this study if the KM prescribed with a personalized manner (termed as personalized Kampo medicine (PKM)) facilitated CTL responses and clinical benefits induced by PPV for aEC patients.

## 2. Patients and Methods

### 2.1. Patients

Patients diagnosed with aEC were eligible for this study. All patients were required to have been diagnosed as Stage III and Stage IV or recurrent at the time of entry. They had to show positive IgG responses to at least 2 of the 31 different vaccine candidate peptides, as reported previously [5–7]. The other inclusion criteria were as follows: an Eastern Cooperative Oncology Group (ECOG) performance status of 0 or 1 at the time of first visit; positive status for the human leukocyte antigen- (HLA-) A2, HLA-A24, or HLA-A3 supertypes (A3, A11, A31, or A33) or the HLA-A26 type; life expectancy of at least 12 weeks; and adequate hematologic, hepatic, and renal function. Exclusion criteria included pulmonary, cardiac, or other systemic diseases; an acute infection; a history of severe allergic reactions; pregnancy or nursing; and other inappropriate conditions for enrollment as judged by clinicians. The protocol was approved by the Kurume University Ethical Committee and registered in the UMIN Clinical Trials Registry (UMIN numbers 1482, 1839, 2984, 6927, 10068, and 11230). All patients were given a full explanation of the protocol and provided their informed consent before enrollment.

### 2.2. Clinical Protocol

This was a phase II study to evaluate the safety, immunological responses, and clinical benefits of PPV in aEC patients. Thirty-one peptides were employed for vaccination [12 peptides for HLA-A2, 14 peptides for HLA-A24, 9 peptides for HLA-A3 supertypes (HLA-A3, HLA-A11, HLA-A31, and HLA-A33), and 4 peptides for HLA-A26] as reported previously [5–7] (Supplementary Table 1 which is available in Supplementary Material available online at http://dx.doi.org/10.1155/2016/5929525). These peptides were prepared under the conditions of Good Manufacturing Practice by the PolyPeptide Laboratories (San Diego, CA) and American Peptide Company (Vista, CA). Peptides for vaccination to an individual patient were selected in consideration of the preexisting host immunity before vaccination, as assessed by the titers of IgG specific to each of the 31 different vaccine candidates [5–7]. A maximum of 4 peptides (3 mg/each peptide), which were selected based on the results of HLA typing and peptide-specific IgG titers, were subcutaneously injected with incomplete Freund's adjuvant (Montanide ISA51; Seppic, Paris, France) once a week for 6 consecutive weeks (UMIN numbers 1482, 1839, and 10068) and every week for 4 weeks followed by administration every 2 weeks for 4 times (UMIN number 2984) and administration every 4 weeks for a total of 4 times (UMIN numbers 6927, 11230), as the 1st cycle. Thereafter, the 4 antigen peptides were reselected according to the titers of peptide-specific IgG followed by the injection. During the PPV, patients were allowed to receive combination therapies (chemotherapies, radiotherapies, or KM). Adverse events were evaluated using the Common Terminology Criteria for Adverse Events version 4.0 throughout the treatment period until a minimum of 28 days after the last dose or until all drug-related adverse events had recovered to baseline or were deemed irreversible. Tumor assessments by computed tomography or magnetic resonance imaging scans were carried out at baseline and after the sixth vaccination and evaluated according to Response Evaluation Criteria In Solid Tumors version 1.1 [5–7].

### 2.3. Combined Therapies

The reagents used for chemotherapy combined with PPV were approved in Japan for the treatment of esophageal cancer. The reagents used for PKM combined with PPV were also approved in Japan for medical use. KM is the Japanese study and adaptation of Traditional Chinese medicine. KM is integrated into the national health care system in Japan. Presently, 148 different KMs have been approved for reimbursement by the Ministry of Health, Labour and Welfare Japan, and 20 of them were used in this study. Details of them were shown in Supplementary Table 2 in which the numbers of each KM and their names have been described as follows: 1: Kakkonto, 14: Hangeshashinto, 15: Orengedokuto, 16: Hangekobokuto, 19: Shoseiryuto, 23: Tokishakuyakusan, 25: Keishibukuryogan, 29: Bakumonto, 41: Hochuekkito, 43: Rikkunshito, 48: Jyuzentaihoto, 68: Shakuyakukanzoto, 89: Jidabokuippo, 90: Seihaito, 100: Daikenchuto 105: Tsudosan, 107: Goshajinkigan, 108: Ninjinyoeito, 125: Keishibukuryogankayokuinin, and 138: Kikyoto. Their formula catalogues are available from Kampo list (http://www.keio-kampo.jp/vc/catalog/formulas/index.html) provided from Keio University, Tokyo, Japan. In addition, general information of KM is available from Wikipedia, the free encyclopedia (https://en.wikipedia.org/wiki/Kampo). Either Kyukichosetsuindaiichikagen or Keppuchikuoto is not yet approved in Japan for medical use but holds potential to facilitate blood circulation of tumor site because of formula catalogues as follows: Keppuchikuoto: tonin 12 g, toki 9 g, shojio 9 g, koka 9 g, sekishaku 6 g, kikoku 6 g, senkyo 5 g, saiko 3 g, kanzo 3 g, goshitu 9 g, and kikyo 5 g; and Kyukichoketsuindaiichikagen: toki 2 g, senkyu 2 g, jio 2 g, byakujyutsu 2 g, bukuryo 2 g, chinpi 2 g, uyaku 2 g, kobushi 2 g, botanpi 2 g, yakumoso 2 g, daiso 2 g, kankyo 1 g, shakanzo 1 g, shakuyaku 3 g, tonin 3 g, koka 2 g, goshitu 2 g, kikoku 2 g, mokko 2 g, engosaku 2 g, and nikkei 1 g. In PKM, several different types of KMs shown above were prescribed in a personalized manner based on the symptoms and laboratory data of each patient. Palliative radiation therapy was also permitted in combination with PPV.

### 2.4. Expression of Vaccine Antigens

The expression levels of the 15 vaccine antigens that code the peptides were examined by immunohistochemical staining in tumor tissues from nonvaccinated esophageal (*n* = 10) cancer patients. Detailed methods including the antibodies used for IHC were previously described [6, 7, 9, 10].

### 2.5. Measurement of CTL, IgG Responses, and Cytokines

CTL activity specific to each of the HLA-matched vaccinated peptides was evaluated by IFN-*γ* ELISPOT assay using PBMCs as reported previously [5–10]. As nonvaccinated peptides, a mixture of virus-derived CTL epitopes (CEF peptides, Mabtech) was provided for the assay. All assays were carried out in triplicate and analyzed with an ELISPOT reader (CTL-ImmunoSpot S5 Series; Cellular Technology Ltd., Shaker Heights, USA). CTL activity was evaluated by the difference between spot numbers in response to the corresponding peptide and those of the control peptide. The cut-off level was set as 10 IFN *γ*-spots per 10^5^ PBMCs. If the spot numbers, in response to the corresponding peptide in postvaccination PBMCs, were more than twofold higher than those in prevaccination PBMCs, the changes were considered to represent positive immune responses, as reported previously [5–10]. The changes were also considered to be positive if the spot numbers that were under 10 in the prevaccination samples became detectable after the vaccination. An IgG response specific to HLA-matched peptides was determined by peptide-specific IgG levels using a Luminex system (Luminex, Austin, TX, USA). The cut-off level of fluorescence intensity unit (FIU) titers was set as 10. If titers of peptide-specific IgG in the postvaccination plasma were more than twofold higher than those in the prevaccination plasma, the increases were considered to be positive immune responses as reported previously [5–10]. The levels of 15 different cytokines including interleukin-6 (IL-6) in plasma before and after 1 cycle of vaccination were examined by ELISA using kits from Life Technologies as reported previously [5–10].

### 2.6. Assessment of Clinical Activity

Preregistration assessments included a detailed medical history, chest and abdominal CT scan, and physical examination. Patients were monitored at each visit by history and physical examinations. CT and routine laboratory studies were performed every 2 to 3 months for efficacy assessments in most cases. Median PFS was defined as the time in months from the first day of peptide vaccination until objective disease progression or death, whichever occurred first. Progression was defined as clinically evaluated progression, radiologic progression, or death as a result of esophageal cancer. Patient data were censored at the time of emigration or disappearance or the day of study cutoff (November 20, 2015). Overall survival (OS) was calculated as the time in months from the first day of peptide vaccination until the date of death or the last date when the patient was known to be alive. Analyses of primary and secondary efficacy endpoints of PFS and OS were based on a full analysis set (FAS) that included all 34 assigned patients who received PPV. Safety was assessed throughout the study by the monitoring of adverse events (assessed according to the National Cancer Institute Common Terminology Criteria for Adverse Events version 4.0 [NCI-CTC Ver. 4]), biochemical laboratory tests, vital signs, and physical examinations.

### 2.7. Statistical Analyses

Time-to-event endpoints were analyzed using the Kaplan–Meier method, and between-treatment comparisons for PFS and OS were conducted using the log-rank test with a two-sided significance level of 5%. Cox proportional hazards analysis was used to calculate hazard ratios (HRs) and 95% confidence intervals (CIs). Student's *t*-test and the chi-square test were used to compare quantitative and categorical variables, respectively. A two-sided significance level of 5% was considered statistically significant. All statistical analyses were conducted using the JMP version 12 (SAS Institute Inc., NC, USA).

## 3. Results

### 3.1. Antigen Expression

**Figure 1:**
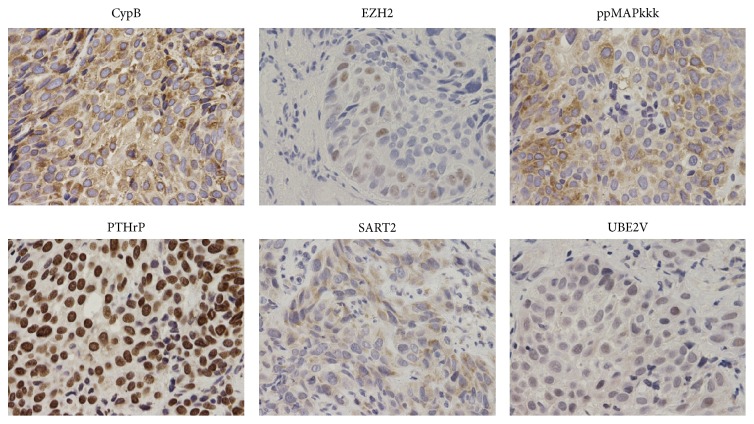
Antigen expression: the expression levels of the 15 vaccine antigens were examined by immunohistochemical staining in tumor tissues from nonvaccinated esophageal squamous cell carcinomas (*n* = 10). Representative results of immunohistochemical staining of 6 antigens are shown.

Because the original tumors of each patient under PPV were not available, we examined the expression of 15 different mother antigens in resected tumors or biopsy samples from nonvaccinated squamous cell carcinoma of the esophagus (*n* = 10). The results showed positivity for SART3 in 10 of 10, HNRPL in 10 of 10, WHSC2 in 9 of 10, EZH2 in 9 of 10, CypB in 9 of 10, EGFR in 8 of 10, UBE2V in 5 of 10, PTHrP in 4 of 10, SART2 in 4 of 10, ppMAPkkk in 3 of 10, MRP3 in 0 of 10, Lck in 0 of 10, PSA in 0 of 10, PAP in 0 of 10, and PSMA in 0 of 10 samples. Some of these results were reported in a recent publication [10]. Representative results for nonreported 6 antigens (CypB, EZH2, ppMAPkkk, PTHrP, SART2, and UBE2V) are shown in Figure 1.

### 3.2. Patients' Characteristics

Thirty-four aEC patients were enrolled in this study between April 2010 and July 2015 as shown in Table 1. Among them, 14 or 20 patients were treated with surgery or radical radiotherapy prior to entry, respectively. Thirty-one patients were previously treated with chemotherapies with 1 (*n* = 7), 2 (*n* = 14), and ≥3 different regimens (*n* = 7), respectively. All but 5 patients were refractory to the available standard therapies. At the time of entry, lymphopenia (88%), anemia (85%), hypoalbuminemia (49%), plasma C-reactive protein (CRP) elevation (46%), abnormal liver functions (46%), and abnormal renal functions (24%) were observed. The vast majority of patients (97%) suffered from various symptoms, including dyspepsia (60%), chest pain (50%), anorexia (43%), cough and/or sputa (37%), nausea (24%), fatigue (24%), constipation (24%), and hoarseness (24%). The results on each patient are shown in Supplementary Table 2.

We previously reported that one of the KMs, JTT, did not significantly affect CTL or IgG responses specific to the vaccine antigens in an open label randomized clinical trial of PPV for patients with advanced pancreatic cancer [8]. JTT had been prescribed for the two patients prior to entering the PPV study without a personalized manner. Therefore, 2 cases who received JTT only were classified as a group without PKM in this retrospective analysis. Under this definition, 23 patients received PKM and PPV (termed as PKM and PPV arm), whereas the remaining 11 patients (9 patients without any KM and 2 patients with JTT alone) did not receive PKM but received PPV (termed as PPV without PKM arm). There were no significant differences between these 23 and 11 patients with regard to age, gender, performance status, histology, clinical stage, previously conducted treatments, numbers of vaccinations, and combined chemotherapy. These results are shown in Table 1. Details of the combination therapies are described in Supplementary Table 2.

As the combination therapy to be used with PPV, 16 patients were eligible for salvage chemotherapy (termed as PPV and chemotherapy arm), whereas the remaining 18 patients were considered ineligible due to potential intolerance (termed as PPV without chemotherapy arm). There were also no significant differences between these 2 arms with regard to age, gender, performance status, histology, clinical stage, previously conducted treatment (data not shown).

### 3.3. Adverse Events

The overall adverse events due to any cause both in PKM and PPV arm (*n* = 23) and in PPV without PKM arm (*n* = 11) are listed in Table 2. The most frequently reported adverse events in PKM and PPV arm were dermatologic reactions at the injection sites (61%), hypoalbuminemia (30%), lymphopenia (30%), and anorexia (26%). In PPV without PKM arm, they were dermatologic reactions at the injection sites (64%), ALP elevation (55%) (*P* < 0.01 versus PPV with PKM arm), AST elevation (45%) (*P* < 0.01), hypoalbuminemia (45%), anemia (36%), ALT elevation (36%) (*P* < 0.01), and GGT elevation (36%) (*P* < 0.01). The mean incidences of adverse events per patient in the former and latter arms were 3.1 (71 in 23 patients) or 5.4 (59 in 11 patients) (*P* = 0.07), respectively, regardless of the fact that the incidence of PPV-related dermatologic reactions at the injection sites did not differ between the two arms. Therefore, after removing the PPV-related skin reactions, the mean incidence of adverse events per patient in the PKM and PPV arm was significantly lower than that in PPV without PKM arm (2.5 versus 4.7, *P* = 0.048). The numbers of severe adverse events (SAE) in PKM with PPV or PPV without PKM arm were 8 or 16, respectively, and all were considered unrelated to the PKM with PPV. The numbers of patients with grades 3, 4, and 5 SAE in PKM with PPV arm were 7, 0, and 1, while those in the PPV without PKM arm were 11, 2, or 3, respectively. The percentage of SAE (8 of 71; 11.3%) among all the adverse events in PKM and PPV arm was significantly lower (*P* = 0.02) than that (16 of 59, 27.1%) in PPV without PKM arm.

The overall adverse events due to any cause both in PPV and chemotherapy (*n* = 16) and in PPV without chemotherapy (*n* = 18) arms are listed in Supplementary Table 3. In PPV and chemotherapy arm, the most frequently reported adverse events were dermatologic reactions at injection sites (88%) (*P* < 0.01 versus PPV without chemotherapy arm), lymphopenia (50%, *P* = 0.01), hypoalbuminemia (38%), anemia (31%), and anorexia (31%). In PPV without chemotherapy arm (*n* = 18), they were dermatologic reactions at injection sites (39%), hypoalbuminemia (33%), anemia (17%), hyponatremia (17%), pneumonitis (17%), and lymphopenia (11%). The mean incidences of adverse events per patient in the former and latter arms were 5.1 (82 in 16 patients) or 2.7 (49 in 18 patients) (*P* < 0.01), respectively. The numbers of SAE in the former and latter arms were 10 and 13, respectively, and one grade 3 lymphopenia was considered related to chemotherapy used for the combination therapy. The numbers of grade 3, 4, or 5 SAE in the former arm was 9, 0, or 1, while that in the latter arm was 9, 2, or 3, respectively.

### 3.4. CTL and IgG Responses to the Vaccine Peptides

**Figure 2:**
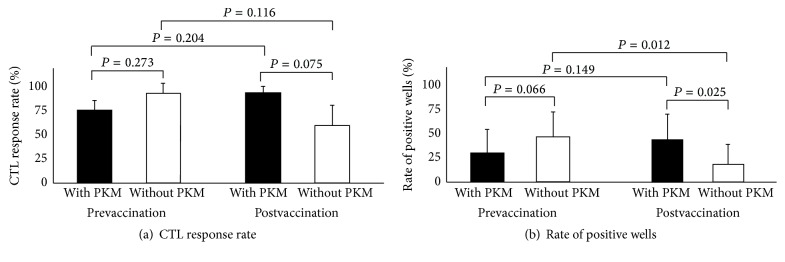
CTL response: CTL responses to the vaccinated peptides in prevaccination PBMCs and in postvaccination (at the end of the first cycle) are shown. (a) Percentage of positive CTL responses among patients tested. (b) Percentage of positive wells among total wells tested.

Lower levels of CTL responses to at least one of the vaccinated peptides were detectable in prevaccination PBMCs from 25 of 32 (78%) patients tested (Supplementary Table 4). Among them, 16 of 22 (73%) patients in PKM and PPV arm or 9 of 10 (90%) patients in PPV without PKM arm showed positive CTL responses, respectively (Figure 2(a)). The numbers of positive wells were 27 of 88 (31%) and 19 of 40 (48%) in the former and latter arms (Figure 2(b)), respectively.

Postvaccination PBMCs at the end of the 1st cycle from 25 patients were provided for CTL assays, but those from the other patients were not available for CTL assays due to the early dropout due to rapid disease progression or low numbers of available samples. Under this limitation, 20 of 25 (80%) patients tested showed augmented CTL responses with 16 of 18 (89%) patients in PKM with PPV arm versus 4 of 7 (57%) patients in PPV without PKM arm (Figure 2(a)). The number of wells showing augmented CTL activity was 30 of 72 (42%) in the former arm, which was significantly higher than that in the latter arm (5 of 28 wells, 18%) (*P* = 0.03) (Figure 2(b)). Twelve of 15 (80%) patients in PPV and chemotherapy arm or 8 of 10 (80%) patients in PPV without chemotherapy arm showed augmented CTL responses. The number of wells showing augmented CTL activity was 23 of 64 (36%) in the former arm, and this percentage was not significantly higher than that in the latter arm (12 of 40 wells, 30%).

CTL responses specific to CEF peptides, a mixture of virus-derived CTL epitopes, in prevaccination PBMCs were detectable in 29 of 32 patients tested, and they were mostly unchanged at the end of the 1st cycle (data not shown). IgG responses specific to the vaccinated peptides were augmented in 13 of 26 (50%) patients tested. Among them, 9 of 19 (47%) patients in PKM and PPV arm or 4 of 7 (57%) patients in PPV without PKM arm showed the augmented IgG responses, respectively. Twelve of 15 (80%) patients in PPV and chemotherapy arm or 8 of 10 (80%) patients in PPV without chemotherapy showed augmented IgG responses, respectively.

Collectively, these results suggested that PKM, but not chemotherapy, facilitated PPV-induced CTL responses specific to the vaccinated peptides, whereas neither PKM nor chemotherapy affected the PPV-induced IgG responses.

### 3.5. Progression-Free Survival and Overall Survival

**Figure 3:**
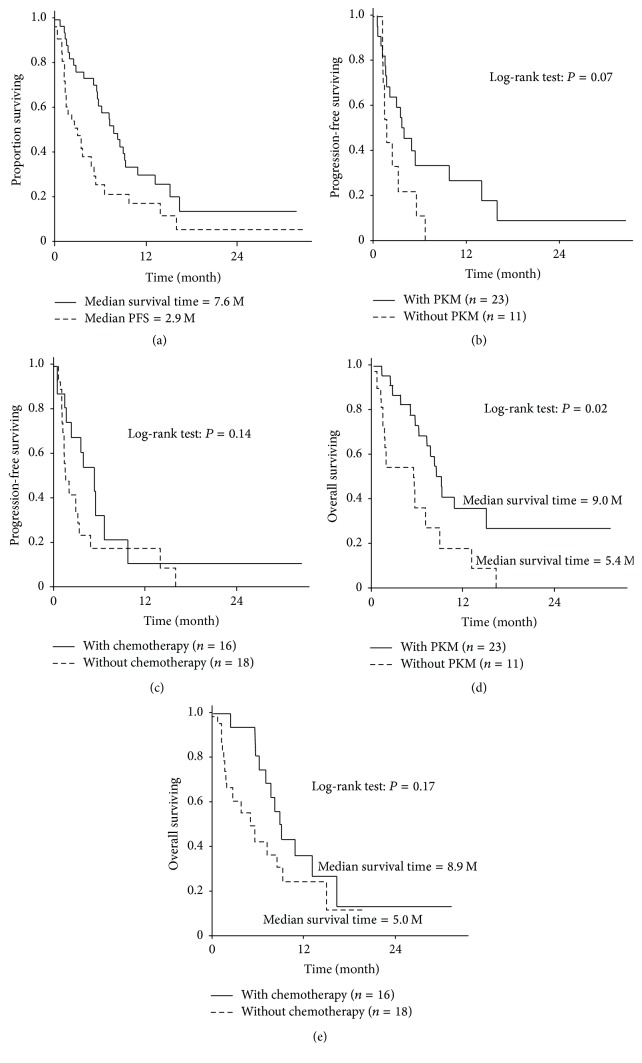
Progression-free survival and overall survival. (a) Median PFS or MST of all 34 patients. (b) PFS of the PPV patients combined with or without PKM. (c) PFS of the PPV patients combined with or without chemotherapy. (d) MST of the PPV patients combined with or without PKM. (e) MST of the PPV patients combined with or without chemotherapy.

The median PFS and MST of all 34 patients from the first vaccination of PPV were 2.9 months (95% confidence interval [CI]: 1.3–5.1) and 7.6 months (95% CI: 5.4–9.2), respectively (Figure 3(a)). Among them, the median PFS for the patients in PKM and PPV (*n* = 23) and in PPV without PKM arms (*n* = 11) was 3.7 (95% CI: 1.3–9.6) and 1.4 (95% CI: 0.9–5.3) months, respectively (*P* = 0.07) (Figure 3(b)). That of the patients in PPV and chemotherapy (*n* = 16) and in PPV without chemotherapy (*n* = 18) arms was 5.1 (95% CI: 1.4–6.5) and 1.4 (95% CI: 0.9–3.2) months, respectively (*P* = 0.14) (Figure 3(c)). The MST of the patients in PKM and PPV and in PPV without PKM arms were 9.0 (95% CI: 6.1–15) and 5.4 (95% CI: 1.1–8.8) months, respectively (*P* = 0.02) (Figure 3(d)). That of the patients in PPV and chemotherapy or in PPV without chemotherapy was 8.9 (95% CI: 6.1–16.2) or 5.0 (95% CI: 1.6–9.2) months, respectively (*P* = 0.17) (Figure 3(e)). These results suggested that PKM, but not chemotherapy, significantly prolonged the OS of aEC patients under PPV.

To better understand the clinical efficacy of the combination therapies, 34 patients were divided into 4 groups: a group treated with all the three (PKM, PPV, and chemotherapy) (*n* = 11); a group receiving PKM and PPV (*n* = 12); a group receiving PPV and chemotherapy (*n* = 5); and a group receiving PPV alone (*n* = 6). The MST values of these groups were 9.0, 8.4, 8.8, and 1.5 months, respectively. Because only 6 cases were deemed intolerant to chemotherapy and given PPV alone, it was difficult to evaluate the clinical benefits of PPV alone for aEC based on our results.

### 3.6. Biomarker Study

**Figure 4:**
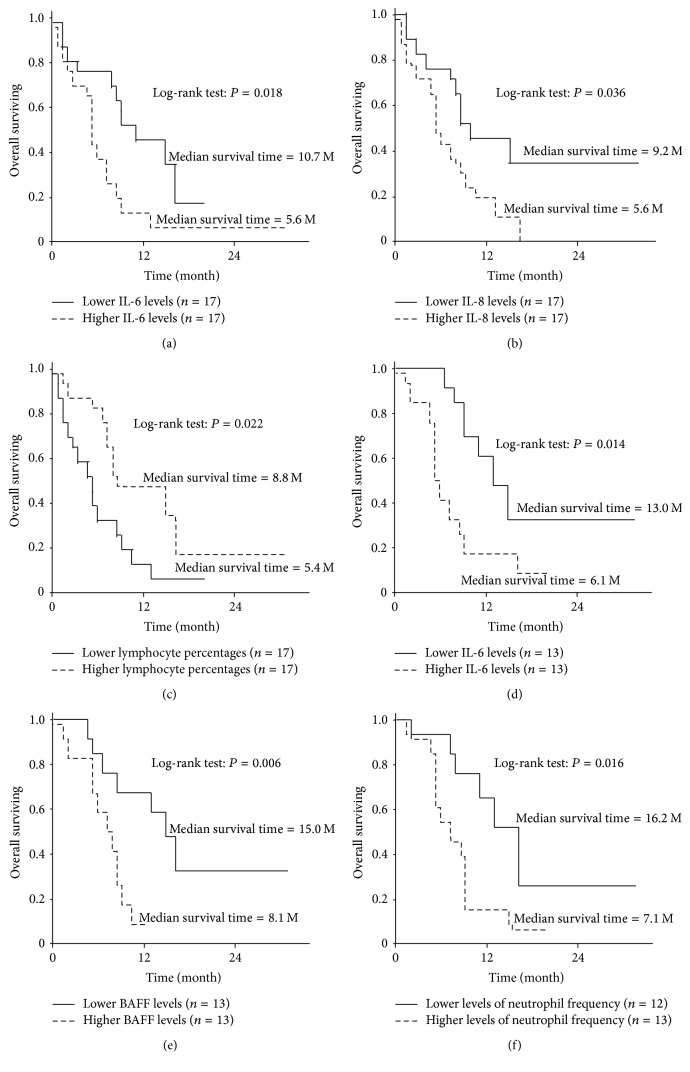
Biomarker study: analysis of biomarkers predictive of OS time using prevaccination samples. Results for IL-6 (a) and IL-8 (b) and the percentages of lymphocytes (c) are shown. Results of biomarker studies prognostic of OS time using postvaccination samples were shown as follows: IL-6 in (d), BAFF in (e), and neutrophil percentage in (f).

We also analyzed biomarkers for OS time using the prevaccination samples. There was no correlation between OS time and either prevaccination CTL activity or IgG responses (data not shown). Higher levels of prevaccination interleukin-6 (IL-6) (median value: 5.66; *P* = 0.02) (Figure 4(a)), interleukin-8 (IL-8) (median value: 4.64; *P* = 0.04) (Figure 4(b)), haptoglobin (Hp) (median value: 1407; *P* = 0.03), and tumor growth factor *β* (median value: 1851; *P* = 0.04) were significantly associated with unfavorable OS of the patients (Table 3). None of the other cytokines tested (IL-1*β*, IL-2, IL-4, IL-5, IL-10, IL-21, GM-CSF, TNF*α*, IFN*γ*, IFN*α*, BAFF, and IP-10) or the neutrophil percentages, numbers of white blood cells, or platelet numbers was associated with the OS time. Lower levels of hemoglobin (Hb) (median value: 11.55; *P* = 0.02) and lower lymphocyte percentages (median value: 21.9%; *P* = 0.02) (Figure 4(c)) were significantly associated with unfavorable OS of the patients.

We then assessed biomarkers for OS time using postvaccination samples obtained at the end of the first cycle. There was no correlation between OS time and either CTL activity or IgG responses (data not shown). In contrast, higher levels of IL-6 (median value: 7.65; *P* = 0.01) (Figure 4(d)), IL-8 (median value: 3.55; *P* = 0.02), or B-cell activating factor belonging to the tumor necrosis factor family (BAFF) (median value: 876; *P* = 0.06) (Figure 4(e)) were significantly associated with unfavorable OS of the patients. In addition, lower lymphocyte percentages (median value: 19.7%; *P* = 0.02) and higher neutrophil percentages (median value: 69.4%; *P* = 0.02) (Figure 4(f)) were significantly associated with unfavorable OS of the patients.

## 4. Discussion

The results of immunohistochemical study showed that 10 of 15 antigens were well expressed, whereas the other 5 antigens were undetectable, which was consistent with the expression profiles of other types of cancers reported previously [6–10]. Based on these results, the peptides derived from 3 prostate-related antigens (PSA, PAP, and PSMA) were chosen only if the IgGs reactive to the other peptides derived from the remaining 12 antigens were not detectable in prevaccination plasma. In contrast, the peptides derived from Lck or MRP3 were chosen when the IgGs reactive to them were detectable in prevaccination plasma, since Lck or MRP3 was highly expressed in the metastatic tumors or tumor cells after chemotherapy, respectively [5–9], and thus negative expression of these two antigens on 10 primary esophageal tumor samples before chemotherapy was expected as reported previously.

We then evaluated the effect of the combination therapy on adverse events. Incidence of adverse events was significantly lower or higher in PKM and PPV arm or in PPV and chemotherapy arm as compared to that of the counter arm, respectively. The lower incidence in PKM and PPV arm could be due to both control various symptoms and normalize abnormal laboratory data by PKM. The higher incidence in PPV and chemotherapy arm could be due to both the higher percentages of skin reactions and lymphopenia by chemotherapy. The patients in PPV and chemotherapy arm received more vaccinations than those receiving PPV without chemotherapy (12 versus 5.5 times, *P* = 0.01), which might in turn be responsible for the higher percentages of skin reactions. All these results suggest that PKM was not only safe but further decreased disease-related adverse events, whereas the chemotherapy induced lymphopenia as expected.

Thirdly, we evaluated the effect of combination therapy on PPV-induced immune responses. A modest level of CTL augmentation was observed in postvaccination PBMCs from the vast majority of the patients, indicating that PPV could be feasible for aEC patients. Notably, the combined PKM facilitated the potent CTL responses to the vaccinated peptides but not to the unvaccinated control peptides. In contrast, the combined chemotherapy did not facilitate potent CTL responses. IgG responses specific to the vaccinated peptides were not affected by either combination. It has been well established that KM possesses various immune-modulating and antitumor properties in animal experiments [11–14]. However, to our knowledge, this could be the first clinical study demonstrating that PKM facilitated CTL responses to the vaccinated peptides in cancer patients undergoing immunotherapy. We previously reported that one of the KMs, juzentaihoto (JTT) that was frequently used for advanced cancer patients, did not significantly affect CTL or IgG responses specific to the vaccine antigens in an open label randomized clinical trial of PPV for advanced pancreatic cancer patients [8]. JTT also did not affect overall survival, although it prevented the deterioration of patients' conditions. JTT had been prescribed for the two patients prior to entering the PPV study without a personalized manner. By these reasons, in this retrospective analysis, the 2 patients who received JTT alone were classified into PPV without PKM arm. Based on the symptoms and laboratory data of each patient, we used several different KM compounds from a pool of more than 100 for individual patients in a personalized manner at each time of visiting to control various symptoms and normalize abnormal laboratory data. The quality of life of the majority of the patients entered in this study was severely disturbed with precachexic status at the time of entry. Subsequently, PKM that could prescribe several different KMs selected in a personalized manner could be more feasible than a single KM in a nonpersonalized manner in order to improve the physical condition of patients and augment their immune responses to the vaccinated peptides. However, the ability of personally tailored KM regimens to promote PPV-induced CTL activity will need to be further investigated in large scale of prospective clinical studies of PPV.

This study showed that PKM, but not chemotherapy, significantly prolonged the OS of aEC patients under PPV. This may be due in part to the ability of PKM to reduce adverse events. It could also be partly related to the fact that PKM facilitated CTL responses specific to the vaccinated peptides. However, it is also too early to draw any definitive conclusions, since this was a retrospective analysis with only 34 patients. In addition, nearly half of patients under PKM and PPV also received the combined chemotherapy. Therefore, this study could not deny the contribution of chemotherapy to the prolongation of overall survival of aEC patients under PPV. Indeed, the MST of the patients with PPV and chemotherapy was somewhat longer than that of the patients with PPV without chemotherapy, although the difference was not statistically significant.

This study also showed that the prevaccination biomarkers for unfavorable OS were anemia, lower lymphocyte percentage, higher levels of haptoglobin, and higher levels of inflammatory cytokines (IL-6, IL-8, and TGF*β*), and the postvaccination biomarkers were IL-6, IL-8, BAFF, lower lymphocyte percentage, and higher neutrophil percentage. We previously reported that these inflammatory cytokines were unfavorable factors in the other types of advanced cancers under PPV [5–9]. However, this study newly showed that lower lymphocyte percentage and higher neutrophil percentage, a typical white blood cell unbalance associated with severe inflammatory responses, were unfavorable factors for advanced cancers under PPV. Anemia, reduced albumin, and reduced platelets were all unfavorable for OS. Taken together, these results indicated that robust inflammation at tumor sites might hamper the OS of aEC patients under PPV.

## 5. Conclusion

PKM could be suitable as one of the treatment modalities combined with PPV for aEC patients because of safety, reduction of the incidence of disease-associated adverse events, and possible enhancement of PPV-induced CTL responses in association of clinical benefits.

## Supplementary Material

Supplementary Table 1 showed symbol for peptide, HLA types, origin protein, position of peptide, and amino acid sequence of 31 different peptide candidates used for personalized peptide vaccination in this study.
Supplementary Table 2 showed the symptoms, laboratory data at the time of first visit, and the combined therapy of each of all patients entered to this study (*n* = 34).
Supplementary Table 3 showed the summary of adverse events of patients under PPV with (*n* = 16) or without chemotherapy (*n* = 18).
Supplementary Table 4 showed the vaccinated peptides, peptide-specific IgG responses and CTL responses to the vaccinated peptides in pre-vaccination and post-vaccination samples from each of all 34 patients.

## Figures and Tables

**Table 1:** Patients' characteristics (*n* = 34).

Parameters	PPV with PKM (*n* = 23)	PPV without PKM (*n* = 11)	*P* value
Age: median (range)	64 (39–82)	60 (43–72)	0.08
Gender: male (female)	18 (5)	9 (2)	0.81
Performance status			
0	18	7	0.37
1	4	3	0.51
2	1	1	0.58
Histology			
Adenocarcinoma	1	1	0.58
Carcinosarcoma	0	1	0.14
Squamous cell carcinoma	21	8	0.15
Small cell carcinoma	0	1	0.14
Neuroendocrine cell carcinoma	1	0	0.48
Clinical stage			
III	3	2	0.69
IV	17	7	0.54
Recurrence	3	2	0.69
Prior surgery	9	5	0.73
Prior radical radiation	15	5	0.27
Previous chemotherapy (regimens)			
0	2	1	0.97
1	5	2	0.81
2	8	6	0.27
≧3	8	2	0.32
Median vaccinations (range)	10 (1–19)	6 (2–21)	0.90
Combination chemotherapy^†^			
None	12	6	0.90
FP (5-FU + CDDP)	1	1	0.58
AMR	1	0	0.48
DCF (DTX + CDDP + 5-FU)	1	0	0.48
TS-1	2	0	0.31
UFT	0	1	0.14
CPA	3	0	0.21
DTX	3	2	0.69
DTX-NPs	0	1	0.14

^†^5-FU: 5-fluorouracil, CDDP: cisplatin, AMR: amrubicin, DTX: *docetaxel*, TS-1: tegafur/gimeracil/oteracil combination, UFT: tegahur-uracil, CPA: cyclophosphamide, and NPs: nanoparticles.

**Table 2:** Toxicities.

	PPV with PKM (*n* = 23)						PPV without PKM (*n* = 11)						*P* value
	G1	G2	G3	G4	G5	Total	G1	G2	G3	G4	G5	Total	
Injection site reaction	12	2				14	6	1				7	0.88
Blood/bone marrow													
Anemia		4				4	1	2	1			4	0.22
Leukocytopenia	1	2				3	2					2	0.69
Neutropenia	1					1						0	0.48
Lymphopenia	1	6				7	1	1	1			3	0.85
Thrombocytopenia	1					1	1					1	0.58
Laboratory^†^													
AST elevation						0	2	1	2			5	<0.01
ALT elevation						0	1		3			4	<0.01
GGT elevation						0	2	2				4	<0.01
ALP elevation						0	4		2			6	<0.01
CRP elevation	1					1	1					1	0.58
D-dimer elevation	1					1						0	0.48
PT (%) decrease		1				1						0	0.48
Creatinine elevation						0		1				1	0.14
Hyperbilirubinemia						0	1			1		2	0.04
Hypoalbuminemia	4	3				7	1	4				5	0.39
Hyponatremia	2					2	1		1			2	0.42
Hyperkalemia	1					1	2					2	0.18
Hyperuricemia						0	1			1		2	0.04
Hyperglycemia	1					1	1					1	0.58
Hyperlipidemia	1					1	1	1				2	0.18
Gastrointestinal													
Anorexia	2		4			6			1			1	0.25
Dysphagia			1		1	2						0	0.31
Diarrhea		1				1						0	0.48
Esophageal stenosis			1			1						0	0.48
Respiratory													
Hoarseness	1					1						0	0.48
Pleural effusion		1				1						0	0.48
Dyspnea	1					1					1	1	0.58
Pneumonitis			1			1					2	2	0.18
Cough	2	1				3						0	0.21
Urinary													
Hematuria	1					1						0	0.48
Tumor pain		1				1	1					1	0.58
Urticaria	1					1						0	0.48
Fracture	1					1						0	0.48
Malaise		2				2						0	0.31
Fever	2					2						0	0.31
Edema limbs	1					1		1				1	0.58

^†^AST: aspartate aminotransferase, ALT: alanine aminotransferase, and GGT: gamma-glutamyl transferase

ALP: alkaline phosphatase.

**Table 3:** Correlation between OS time and humoral factors in either pre- or postvaccination samples.

Factor^†^	Prevaccination		Postvaccination	
	Median (pg/mL)	Log-rank test (*P*)	Median (pg/mL)	Log-rank test (*P*)
TGF-*β*	1851	0.0402	1795	0.7702
BAFF	928	0.0613	876	0.006
IL-21	3.654	0.7432	2.869	0.7883
IP-10	146	0.0566	203	0.2412
Hp	1407	0.0287	1128	0.1226
IL-1*β*	1.032	0.4875	1.061	0.6922
IL-10	1.517	0.9446	1.681	0.7752
IL-6	5.656	0.0181	7.652	0.0142
GM-CSF	0.481	0.7079	0.506	0.6450
IL-5	0.761	0.9932	0.761	0.1527
IFN-*γ*	1.609	0.716	1.638	0.6320
TNF-*α*	1.930	0.1800	1.998	0.2123
IL-2	0.503	0.9772	0.536	0.8641
IL-4	1.241	0.6980	1.355	0.5126
IL-8	4.644	0.0355	3.548	0.0178

^†^TGF: transforming growth factor beta, BAFF: B-cell activating factor belonging to the tumor necrosis factor family, IL: interleukin, IP: interferon-inducible protein, Hp: haptoglobin, GM-CSF: granulocyte macrophage colony-stimulating factor, and TNF: tumor necrosis factor.

## Abbreviations

EC: Esophageal cancer
PPV: Personalized peptide vaccination
PKM: Personalized Kampo medicine
OS: Overall survival
HLA: Human leukocyte antigen
NCI-CTC: National Cancer Institute Common Terminology Criteria for Adverse Events
CTL: Cytotoxic T lymphocyte
IFN: Interferon
PBMCs: Peripheral blood mononuclear cells
SAE: Severe adverse events
PFS: Progression-free survival
MST: Median survival time.
