# Supplementary material for: Personalized Kampo Medicine Facilitated Both Cytotoxic T Lymphocyte Response and Clinical Benefits Induced by Personalized Peptide Vaccination for Advanced Esophageal Cancer
Source: Evid Based Complement Alternat Med. 2016 Sep 15;2016:5929525. doi: 10.1155/2016/5929525 (PMC5040795; doi:10.1155/2016/5929525)
Supplement: Supplementary file 1 — Supplementary Table 1 showed symbol for peptide, HLA types, origin protein, position of peptide, and amino acid sequence of 31 different peptide candidates used for personalized peptide vaccination in this study. Supplementary Table 2 showed the symptoms, laboratory data at the time of first visit, and the combined therapy of each of all patients entered to this study (n = 34). Supplementary Table 3 showed the summary of adverse events of patients under PPV with (n = 16) or without chemotherapy (n = 18). Supplementary Table 4 showed the vaccinated peptides, peptide-specific IgG responses and CTL responses to the vaccinated peptides in pre-vaccination and post-vaccination samples from each of all 34 patients. [file 5929525.f1.docx]

**Supplemental table 1: Peptide candidates for personalized peptide vaccination**

| Symbol for peptide | HLA type | Origin protein | Position of peptide | Amino acid sequence |
| --- | --- | --- | --- | --- |
| CypB-129 | A2，A3sup | Cyclophilin B | 129-138 | KLKHYGPGWV |
| Lck-246 | A2 | p56 lck | 246-254 | KLVERLGAA |
| Lck-422 | A2，A3sup | p56 lck | 422-430 | DVWSFGILL |
| MAP-432 | A2，A26 | ppMAPkkk | 432-440 | DLLSHAFFA |
| WHSC2-103 | A2，A3sup，A26 | WHSC2 | 103-111 | ASLDSDPWV |
| HNRPL-501 | A2，A26 | HNRPL | 501-510 | NVLHFFNAPL |
| UBE-43 | A2 | UBE2V | 43-51 | RLQEWCSVI |
| UBE-85 | A2 | UBE2V | 85-93 | LIADFLSGL |
| WHSC2-141 | A2 | WHSC2 | 141-149 | ILGELREKV |
| HNRPL-140 | A2 | HNRPL | 140-148 | ALVEFEDVL |
| SART3-302 | A2 | SART3 | 302-310 | LLQAEAPRL |
| SART3-309 | A2 | SART3 | 309-317 | RLAEYQAYI |
| SART2-93 | A24 | SART2 | 93-101 | DYSARWNEI |
| SART3-109 | A24，A3sup，A26 | SART3 | 109-118 | VYDYNCHVDL |
| Lck-208 | A24 | p56 lck | 208-216 | HYTNASDGL |
| PAP-213 | A24 | PAP | 213-221 | LYCESVHNF |
| PSA-248 | A24 | PSA | 248-257 | HYRKWIKDTI |
| EGFR-800 | A24 | EGF-R | 800-809 | DYVREHKDNI |
| MRP3-503 | A24 | MRP3 | 503-511 | LYAWEPSFL |
| MRP3-1293 | A24 | MRP3 | 1293-1302 | NYSVRYRPGL |
| SART2-161 | A24 | SART2 | 161-169 | AYDFLYNYL |
| Lck-486 | A24 | p56 lck | 486-494 | TFDYLRSVL |
| Lck-488 | A24 | p56 lck | 488-497 | DYLRSVLEDF |
| PSMA-624 | A24 | PSMA | 624-632 | TYSVSFDSL |
| EZH2-735 | A24 | EZH2 | 735-743 | KYVGIEREM |
| PTHrP-102 | A24 | PTHrP | 102-111 | RYLTQETNKV |
| SART3-511 | A3sup | SART3 | 511-519 | WLEYYNLER |
| SART3-734 | A3sup | SART3 | 734-742 | QIRPIFSNR |
| Lck-90 | A3sup | p56 lck | 90-99 | ILEQSGEWWK |
| Lck-449 | A3sup | p56 lck | 449-458 | VIQNLERGYR |
| PAP-248 | A3sup | PAP | 248-257 | GIHKQKEKSR |

A3sup = HLA-A3 supertype (A3, A11, A31, and A33). HLA = human leukocyte antigen.

Note: The safety and immunological effects of these 31 peptides had been confirmed in previous clinical trials^10^ and all peptides were prepared under conditions of Good Manufacturing Practice using a Multiple Peptide System (San Diego, CA

**Supplemental Table 2: Symptoms and laboratory data at the time of first visit, and the combined therapy (n=34)**

Combined therapy

Patient No. Symptoms at the first visit Laboratory data at the first visit ―――――――――――――――――――――――――――――――――

Kampo medicine^✝^ Chemotherapy^‡^

1 Exertional dyspnea, Expectoration, Fatigue, Pain Hypoalbuminemia, Anemia, Atelectasis UFT

2 Chest-tightness, Dysphagia, Fatigue, Anorexia, Pain Hypoalbuminemia, Anemia, Leukocytosis

Lymphopenia, Hepatopathy, Nephropathy

3 Hypoalbuminemia, Anemia, Leukocytosis Anemia, Lymphopenia, Hepatopathy 5-FU+CDDP

Lymphopenia, Hepatopathy, Nephropathy CRP elevation

4 Dysphagia, Hyperlipidemia Anemia, Leukocytopenia, Lymphopenia DTX-NPs, 5-FU

5 Dysphagia, Pain, Anorexia, Insomnia Anemia, Lymphopenia 48

6 Constipation, Nephropathy Hypoalbuminemia, Anemia, Lymphopenia 48

7 Anorexia, Paresthesia, Bloating Anemia, Lymphopenia, Dyslipidemia

Nephropathy

8 Chest tightness, Dysphagia, Fatigue, Hoarseness Hypoalbuminemia, Anemia, Leukocytosis 29, 108, 138

Lymphopenia, Nephropathy, CRP elevation

9 Chest tightness, Dysphagia, Fatigue, Anorexia, Pain Hypoalbuminemia, Leukocytosis, Anemia

Lymphopenia, Dyslipidemia

10 Nausea, Hoarseness, Expectoration, Esophageal stenosis Anemia, Leukocytopenia, Thrombocytopenia 14, 16, 43, 48 CPA

Lymphopenia, Dyslipidemia

11 Dysphagia, Exertional dyspnea, Skin infiltration, Pain Anemia, Thrombocytopenia, Lymphopenia 43, 108 TS-1

Dyslipidemia

12 Fatigue, Anorexia, Cough Hypoalbuminemia, Anemia, Lymphopenia 29, 43, 90, 108

13 Paronychia, Pain Anemia, Lymphopenia

14 Hoarseness, Cough, Pain Anemia, Lymphopenia, Dyslipidemia 29, 25, 48 DTX, CPA

**Supplemental Table 2: Continued.**

Combined therapy

Patient No. Symptoms at the first visit Laboratory data at the first visit ――――――――――――――――――――――――

Kampo medicine^✝^ Chemotherapy^‡^

15 Diarrhea, Anorexia, Fatigue Hypoalbuminemia, Anemia, Leukocytopenia 48, 100 DTX

Nephropathy

16 Insomnia, Edema, Constipation, Blood stasis Hypoalbuminemia, Lymphopenia, Dyslipidemia 25, 41 CPA

17 Dysphagia, Anorexia, Fatigue, Pain, Cough Anemia, Lymphopenia, CRP elevation 14, 25, 41, 43

18 Dysphagia, Nephropathy Eosinophilia, Lymphopenia, CRP elevation kyukichosetsu DTX

indaiichikagen

19 Anorexia, skin infiltration, Pain, Hypoalbuminemia, anemia, Lymphopenia 1, 25, 29, 41, 68, 89, 105

CRP elevation 125, keppuchikuoto

20 Rash, Dysphagia Anemia, Lymphopenia 23, 48, 100

21 Constipation, Diarrhea Anemia, Lymphopenia 16, 23, 48, 15

22 Pain, Esophageal stenosis, Anorexia, Constipation Hypoalbuminemia, Anemia, Lymphopenia 14, 23, 25, 41

Hepatopathy, CRP elevation

23 Dysphagia, Anorexia, Dysgeusia, Stomatitis, Nycturia Anemia, Nephropathy, CRP elevation 107, 25, 14, 43, 105, 48 TS-1

Pain

24 Nausea, Tremor, Anorexia, Cough, Expectoration, Pain Hypoalbuminemia, Anemia, Lymphopenia 16, 19, 25, 43, 48, 90

Hepatopathy, CRP elevation 105, 125

25 Fever, Stomatitis, Epigastric distress, Anorexia, Pain Hypoalbuminemia, Anemia, Lymphopenia 25, 43, 48, 105

Dehydration CRP elevation, Hyperglycemia

26 Dysphagia, Exertional dyspnea, Pain, Anorexia Anemia, Lymphopenia, CRP elevation 14, 25, 90, 105

Expectoration ALP elevation

27 Hoarseness, Constipation, Nycturia, Pain Leukocytopenia, Lymphopenia, Dyslipidemia 14, 25, 29, 41, 48, 105

28 Dysphagia, Anorexia, Fatigue, Constipation, Pain Anemia, Lymphopenia, CRP elevation 14, 16, 25, 29, 48, 105

Dyslipidemia

**Supplemental Table 2: Continued**.

Combined therapy

Patient No. Symptoms at the first visit Laboratory data at the first visit ――――――――――――――――――――――――

Kampo medicine^✝^ Chemotherapy^‡^

29 Anorexia, Hepatopathy, Nephropathy Hypoalbuminemia, Anemia, Lymphopenia DTX

30 Expectoration, Cough, Anorexia, Constipation Hypoalbuminemia, Anemia DTX

31 Hoarseness, Exertional dyspnea, Cough, Odynophagia Anemia, Lymphopenia, CRP elevation 16, 23, 25, 48, 105, 107 CDDP+5-FU+DTX

108, keppuchikuoto

32 Pain, Anorexia, Expectoration, Cough, Constipation Hypoalbuminemia, Anemia, Leukocytopenia 16, 25, 68, 105, 108 AMR, CBDCA, DTX

Hepatopathy keppuchikuoto

33 Pale complexion, Cough, Pain, Exertional dyspnea Hypoalbuminemia, Anemia, Lymphopenia 23, 41, 48, 105

CRP elevation

34 Dysphagia, Skin infiltration, Pain, Hypoalbuminemia, Anemia, Lymphopenia 14, 16, 23, 25, 29, 41 CDDP+5-FU

Hepatopathy 105, 108, keppuchikuoto

✝Numbers indicated the name of extract granules, and their names were as follows; 1: Kakkonto, 14: Hangeshashinto, 15: Orengedokuto, 16: Hangekobokuto,19: Shoseiryuto, 23: Tokishakuyakusan ,25: Keishibukuryogan, 29: Bakumonto, 41: Hochuekito, 43: Rikkunshito, 48: Jyuzentaihoto, 68: Shakuyakukanzoto, 89: Jidabokuippo, 90: Seihaito, 100: Daikenchuto 105: Tsudosan, 107: Goshajinkigan, 108: Ninjinyoeito, 125: Keishibukuryogankayokuinin, 138: Kikyoto. Each formula catalogue is available from Kampo list (<http://www.keio-kampo.jp/vc/catalog/formulas/index.html>) provided from Keio University (Tokyo Japan). In addition, general information of KM is available from Wikipedia, the free encyclopedia (<https://en.wikipedia.org/wiki/Kampo>). Two exceptions among KMs used in this study were kyukichosetsuindaiichikagen and keppuchikuoto. Both of them have potential to promote blood circulation of tumors since of their formula catalogues as follows; Keppuchikuoto : tonin12g, toki 9g, shojio 9g, koka 9g, sekishaku 6g, kikoku 6g, senkyo5g, saiko 3g, kanzo 3g, goshitu9g, kikyo 5g,; kyukichoketsuindaiichikagen: toki 2g, senkyu 2g, jio 2g, byakujyutsu 2g, bukuryo 2g, chinpi 2g, uyaku 2g, kobushi 2g, botanpi 2g, yakumoso 2g, daiso 2g, kankyo 1g, shakanzo 1g, shakuyaku 3g, tonin 3g, koka 2g, goshitu 2g, kikoku 2g, mokko 2g, engosaku 2g, nikkei 1g.

‡UFT: tegafur-uracil, 5-FU: 5-fluorouracil, CDDP: cisplatin, DTX: docetaxel*,* NPs; nanoparticles, CPA: cyclophosphamide, TS-1: S1-tegafur-xxonate combination AMR: amrubicin, CBDCA: carboplatin

**Supplemental Table 3: Adverse events of patients under PPV with or without chemotherapy**

PPV with chemotherapy (n=16) PPV without chemotherapy (n=18) *P* value

G1　G2　G3　G4　G5 Total 　　　 G1　G2　G3　G4　G5 Total

Injection site reaction 12 2　 　　 14 6　　1 7 < 0.01

Blood/Bone Marrow

Anemia 1　　3 1 　 5 　　3　　 　 3 0.32

Leukocytopenia 3　　1 4 1 　 1 0.11

Neutropenia 1 　 1 　 0 0.28

Lymphopenia 3　　4 1 　 8 　　 2　 　 2 0.01

Thrombocytopenia 1 　 1 1 　 1 0.93

Laboratory^†^

AST elevation 2　 1 1 4 　　 　　1 　 1 0.11

ALT elevation 1　 2 　 3 　　　　 1 　 1 0.23

GGT elevation 2 2 　 4 　　　　 0 0.02

ALP elevation 3 1 　 4 1　　1　　 　 2 0.29

CRP elevation 1 　 1 1 　 1 0.93

D-dimer elevation 1 　 1 0 0.28

PT (%) decrease 　　 1 　 1 0 0.28

Creatinine elevation 1 1 　　 0 0.28

Hyperbilirubinemia 　 0 1 1 2 0.17

Hypoalbuminemia 3　　3 　 6 2　　4　　 　 6 0.80

Hyponatremia 1 　 1 2　　　　 1 　 3 0.35

Hyperkalemia 3 　 3 　 0 0.05

Hyperuricemia 1 　 1 　　 1 1 0.93

Hyperglycemia 1 　 1 1 　 1 0.93

Hyperlipidemia 2 　 2 　　1 　 1 0.48

Gastrointestinal

Anorexia 2　　　　3 　 5 　　 　　2 　 2 0.15

Dysphagia 　　　　 1 1 1 　 1 0.93

Diarrhea 　　 1 　 1 0 0.28

Esophageal stenosis 　 0 1 　 1 0.34

Respiratory

Hoarseness 1 　 1 0 0.28

Pleural effusion 　　 1 　 1 0 0.28

Dyspnea 1 　 1 　 1 1 0.93

Pneumonitis 　　　　 　 0 　　1　　2 　 3 0.09

Cough 1　　1 　 2 1　　 　　 　 1 0.48

Urinary

Hematuria 1 　 1 0 0.28

**Supplemental Table 3: Continued.**

PPV with chemotherapy (n=16) PPV without chemotherapy (n=18) *P* value

G1　G2　G3　G4　G5 Total 　　　 G1　G2　G3　G4　G5 Total

Tumor pain 　　 　 0 1 1 　 2 0.17

Urticaria 　 0 1 1 0.34

Fracture 1 　 1 0 0.28

Malaise 　　 2 　 2 　　　　 0 0.12

Fever 　 0 2 　　　　 　 2 0.17

Edema limbs 　 0 1　　 1 　 2 0.17

†AST; aspartate aminotransferase, ALT; alanine aminotransferase, GGT; gamma-glutamyl transferase

ALP; alkaline phosphatase, CRP; C-reactive protein, PT; Prothrombin time

**Supplementary Table.4: Immune responses to the vaccinated peptides**

Patient No. Peptide IgG responses（FIU^✝^） CTL (IFNγ spots/10^5^ cells)

Pre 1cycle Pre 1 cycle

1 Lck-486 1704 2069 ND^‡^ ND

EZH2-735 29 ND 2 ND

SART3-109 3848 6625 46 63

HNRPL-501 798 751 17 ND

2 Lck-422 36 NA^§^ ND NA

HNRPL-501 262 NA 42 NA

Lck-486 92 NA 79 NA

PSMA-624 67 NA ND NA

3 EGF-R-800 209 190 175 228

EZH2-735 86 95 338 146

SART3-109 100 105 148 122

HNRPL-501 159 323^#^ ND 29

4 Lck-246 271 285 19 37

UBE2V-43 198 188 109 ND

Lck-449 19 19 ND 570

CypB-129 257 237 96 ND

5 Lck-208 17 NA 27 NA

MRP3-503 13 NA ND NA

MRP3-1293 18 NA 34 NA

Lck-486 56 NA 81 NA

6 Lck-208 118 123 ND ND

Lck-486 148 133 ND ND

Lck-449 129 115 ND 309

CypB-129 382 259 ND ND

7 SART3-93 23 NA NA NA

Lck-488 19 NA NA NA

SART3-109 547 NA NA NA

SART3-511 21 NA NA NA

8 SART2-93 2663 2261 NA NA

PSA-248 166 265 NA NA

SART3-734 111 49 NA NA

Lck-90 20 63 NA NA

**Supplementary Table.4　 Immune responses (continued)**

Patient No. Peptide IgG responses（FIU^✝^） CTL (IFNγ spots/10^5^ cells)

Pre 1cycle Pre 1 cycle

9 SART3-734 223 776 ND ND

CypB-129 2775 7775 ND ND

ppMAPkkk-432 289 304 ND ND

WHSC2-103 169 177 81 ND

10 Lck-246 31 32 ND 111

Lck-422 69 700 ND 270

SART3-109 74 100 ND ND

SART3-734 1154 1224 10 ND

11 SART2-93 110 92 78 2

SART3-109 419 604 45 7

EGF-R-800 431 335 ND 36

Lck-488 57 50 ND 48

12 WHSC2-141 160 9725 31 ND

SART3-302 269 774 24 656

SART3-109 36 39 ND 49

WHSC2-103 116 103 ND 59

13 SART3-511 188 NA 15 NA

SART3-734 2537 NA ND NA

ppMAPkkk-432 364 NA 37 NA

WHSC2-103 12 NA ND NA

14 SART3-511 74 72 ND ND

Lck-90 47 42 ND ND

PAP-248 274 332 112 9

WHSC2-103 102 117 ND ND

15 SART2-93 45 50 ND ND

SART3-109 25 16 ND 16

Lck-486 78 111 5 181

Lck-488 39 42 ND ND

16 Lck-246 668 NA ND NA

WHSC2-103 93 NA ND NA

PAP-213 2072 NA ND NA

Lck-488 673 NA ND NA

**Supplementary Table.4　 Immune responses (continued)**

Patient No. Peptide IgG responses（FIU^✝^） CTL (IFNγ spots/10^5^ cells)

Pre 1cycle Pre 1 cycle

17 SART2-93 38 NA 4 NA

PSA-248 26 NA ND NA

Lck-486 23 NA ND NA

Lck-488 32 NA 34 NA

18 Lck-246 666 437 20 94

ppMAPkkk-432 191 113 23 117

UBE2V-43 73 57 ND 35

SART3-302 6470 4282 ND 56

19 Lck-246 193 164 1176 93

ppMAPkkk-432 204 178 1004 ND

WHSC2-103 186 172 137 26

Lck-486 283 268 ND 10

20 Lck-246 141 304 ND ND

WHSC2-141 45 243 ND ND

SART3-302 64 4046 ND 115

SART2-93 64 73 27 14

21 SART2-93 823 801 14 5

EGF-R-800 1343 1306 14 ND

Lck-488 78 84 ND 3

SART3-734 53 52 6 ND

22 Lck-422 123 156 50 432

HNRPL-140 256 8927 ND 47

SART3-511 1887 1557 ND ND

Lck-90 243 305 ND ND

23 PAP-213 48 141 ND 1244

Lck-486 29 64 ND 413

SART3-734 3200 2788 ND ND

PAP-248 53 163 2 ND

24 SART3-109 412 94 ND 85

SART3-734 4470 5412 ND 26

Lck-90 222 3771 ND ND

WHSC2-103 1917 1716 ND 22

**Supplementary Table.4　 Immune responses (continued)**

Patient No. Peptide IgG responses（FIU^✝^） CTL (IFNγ spots/10^5^ cells)

Pre 1cycle Pre 1 cycle

25 SART2-93 257 NA 71 NA

Lck-486 174 NA ND NA

PTHrP-102 482 NA ND NA

ppMAPkkk-432 230 NA 44 NA

26 SART2-93 948 695 ND 11

Lck-208 386 2489 21 8

Lck-488 621 666 9 22

SART3-734 20118 18849 ND ND

27 SART3-302 577 1194 ND ND

SART2-93 250 256 39 29

Lck-486 96 92 ND 4

Lck-488 73 76 ND 68

28 SART3-511 26 47 ND ND

Lck-90 12 13 ND ND

PAP-248 26 28 ND ND

CypB-129 15 20 ND 48

29 SART3-109 2348 2625 143 179

Lck-488 215 121 226 ND

SART3-734 4742 5688 ND ND

Lck-90 104 1001 ND ND

30 SART2-93 109 124 ND ND

SART3-109 2649 9451 ND 98

Lck-486 24 37 ND 35

Lck-488 77 56 41 ND

31 CypB-129 71 52 85 18

ppMAPkkk-432 129 99 102 ND

HNRPL-501 148 105 21 56

SART3-302 2704 2284 ND 19

32 ppMAPkkk-432 22 23 ND 4

WHSC2-103 14 12 16 ND

WHSC2-141 19 37 ND 12

SART3-309 15 ND 38 19

**Supplementary Table.4　 Immune responses (continued)**

Patient No. Peptide IgG responses（FIU^✝^） CTL (IFNγ spots/10^5^ cells)

Pre 1cycle Pre 1 cycle

33 SART2-93 31 NA ND NA

Lck-486 41 NA 14 NA

Lck-488 46 NA 53 NA

ppMAPkkk-432 43 NA ND NA

34 Lck-488 30 37 ND 134

SART3-511 352 330 ND ND

SART3-734 6861 9282 ND ND

WHSC2-103 1113 1109 ND ND

✝FIU: fluorescence intensity units, ‡ND: not detectable, §NA: not available for assay

#A box indicated that the IgG response or CTL activity was evaluated as augmented. Details were shown in Patients and methods section.
